# Review of the limitations and potential empirical improvements of the parametric group method of data handling for rainfall modelling

**DOI:** 10.1007/s11356-022-23194-3

**Published:** 2022-10-10

**Authors:** Ronald William Lake, Saeed Shaeri, STMLD Senevirathna

**Affiliations:** 1 School of Computing, Mathematics and Engineering, Charles Sturt University, Bathurst, New South Wales Australia; 2 Gulbali Institute for Agriculture, Water and Environment, Charles Sturt University, Albury, NSW 2640 Australia

**Keywords:** Ensemble empirical mode decomposition, GMDH, Machine learning, Rainfall modelling, Unscented Kalman filter, Wavelet transform

## Abstract

This study furthers the utilisation of the parametric group method of data handling (GMDH) in assessing the possibility of rainfall modelling and prediction, using publicly available temperature and rainfall data. In using ordinary GMDH approaches, the modelling is inconclusive with no clear consistency demonstrated through coefficients of determination and analysis of variance. Hence, an empirical assessment has been undertaken to provide an explanation of the inconsistency. In doing so, state variable distribution, their classification within the fuzzy context, and the need to integrate the principle of incompatibility into the GMDH modelling format are all assessed. The mathematical foundations of GMDH are discussed within the heuristic framework of data partitioning, partial description synthesis, the limitations of the least-squares coefficient of determination, incompleteness theorem, and the necessity for an external criterion in the selection procedure for polynomials. Methods for modelling improvement include the potential for hybridisation with least square support vector machines (LSSVM), the application of filters for parameter estimation, and the combination with signal processing techniques, ensemble empirical mode decomposition (EEMD), wavelet transformation (WT), and wavelet packet transformation (WPT). These have been investigated in addition to the implementation of enhanced GMDH (eGMDH) and fuzzy GMDH (FGMDH). The inclusion of exogenous data and its application within the GMDH modelling paradigm are also discussed. The study concludes with recommendations to enhance the potential for future rainfall modelling study success using parametric GMDH.

**Supplementary Information:**

The online version contains supplementary material available at 10.1007/s11356-022-23194-3.

## Introduction

Rainfall trend analysis is an active research area that includes environmental, agricultural, and engineering studies. Reviewing historical rainfall data that extends back for over decades without discontinuities provides an opportunity for trend identification that may point to a changing climate. The Intergovernmental Panel on Climate Change (IPCC 2014) advises that statistically significant increases in heavy rainfall events have occurred since 1951 across more regions than the converse.

In Australia, the Commonwealth Scientific and Industrial Research Organisation (CSIRO) and the Bureau of Meteorology (BoM) released the State of Climate Report (CSIRO and BoM 2020), indicating that there has been an increase in the intensity of heavy rainfall events over the past 25 years. Based on that report, extreme rainfall events up to and including 60-min duration have also increased in frequency by at least 10% across different regions of the country (CSIRO and BoM 2020, p. 8). Such short-duration events impose the added potential of flash flooding, placing communities at risk. Cashen (2011) quantified the effects of climate change being superimposed over the Australian climate influences of El Niño Southern Oscillation (ENSO) and the Indian Ocean Dipole (IOD). Southern Oscillation in its two forms (i.e., La Niña delivering an increase in rainfall, and El Niño delivering a reduction in rainfall), combined with positive IOD (i.e., rainfall decrease) or negative IOD (i.e., rainfall increase), oscillate about the typical rainfall trend (Cashen 2011).

There is considerable rainfall variability across the Central West of New South Wales region, which is a result of complex interactions between weather patterns, large-scale climate influences such as ENSO, the topography of the Blue Mountains and the Great Dividing Range, coupled with the circa 80-km straight line distance from the East Coast of NSW (Office of Environment and Heritage 2014). Much of the Central West experiences annual mean precipitation within the 400–800 mm range. This is in contrast to the far western plains, which receive only around 400 mm per year (Office of Environment and Heritage 2014).

As a means of attempting to quantify the existence of trends in rainfall data to predict future rainfall events, a selection of six local government areas (LGAs) within the Central West of NSW, Australia, was chosen for this research. The LGAs are Forbes, Lachlan, Bland, Parkes, Cowra, and Weddin, where the historical rainfall datasets recorded by the BoM (2020) are publicly available. Some of the adjoining LGAs (such as Cabonne) are purposely excluded as their catchment characteristics are quite disparate, influencing noticeable meteorological differences (for instance, Cabonne’s rainfall ranges within 800–1200 mm per year). The choice for exclusion is also supported by the Central West and Orana—climate change snapshot (2014).

In 2020–2021, the first author studied and analysed (Lake 2021) the variation of monthly temperature and rainfall data using machine learning and polynomial neural network (PNN), specifically the group method of data handling (GMDH) to investigate their relationship and correlation. To the authors’ knowledge, no other published studies have used GMDH within a regional Australian context for time series rainfall and temperature trend analysis. Accordingly, GMDH Shell version 3 (GMDH 2022) has been utilised which is machine learning software, available for a windows 10 operating system (with user access to influence regressor distribution or external criterion not permitted). The modelling results illustrated a noticeable disparity between the accuracy of the temperature modelling when compared with the rainfall modelling using factors such as coefficients of determination. Cox et al. (2019) also reported a similar outcome and detailed it as intrinsically noisy, featuring a large scatter with no obvious trend. However, due to the capabilities offered by such methods (e.g., Aghelpour and Varshavian 2020; Nguyen et al. 2019), the authors committed to expanding their approach and investigating the potential for improvement of GMDH.

To overcome the modelling issues and achieve the benefits of the GMDH approach, this paper aims to study an established collection of methods that, when combined with GMDH, deliver modelling improvement. Many of these improvements to GMDH were not utilised for rainfall modelling, but the results illustrate the potential if applied within the rainfall domain. Hence, this paper brings together these methods of improvement that, to the author’s knowledge, have not been presented collectively in a single study. Each section details a mathematical background and guidance for its implementation in a rigorous and informative manner utilising appendices to illustrate the mathematics. The composition of parametric GMDH is introduced together with the formation of combinatorial algorithms (COMBI) and multilayered iterative algorithms (MIA) (Madala and Ivakhnenko 1994). Least square support vector machines (LSSVM), the application of unscented Kalman filters (UKF) for polynomial parameter determination, and three signal processing techniques were investigated for the hybridisation with GMDH. Variations of GMDH are also discussed within the context of enhanced and fuzzy prior to suggesting additional methods that may prove beneficial for modelling accuracy. It should be noted that within this paper the term standard GMDH applies to the COMBI and MIA algorithms, while parametric GMDH is a single-layer network of neurons.

## Analysis of parametric GMDH

GMDH is machine learning, a branch of artificial intelligence (Wakefield 2021). It was introduced in 1966 by Dr A. G. Ivakhnenko as an algorithm that would allow the development of high-order regression-type polynomials (Farlow 1981). In designing GMDH, Ivakhnenko employed a heuristic and perceptron approach, the latter being a feature of artificial neural networks (ANN) (Anastasakis and Mort 2001). GMDH algorithms are grouped broadly into two categories: parametric and non-parametric. COMBI and MIA fall within the former category, where the input data are either exact or possess noise of low variance (Anastasakis and Mort 2001). These algorithms model the full range of “possible input variable combinations”, selecting the best model that has been generated from the complete set of models according to the external criterion (Anastasakis and Mort 2001, p. 4). Ivakhnenko et al. (1983) define COMBI algorithms as a complete mathematical inductive method, as no potential models will be passed prior to consideration.

COMBI algorithms organise the models through gradual term increments from 1 to *m*, where *m* is the number of arguments, while the external criterion will specify the optimum solution that exists between models that exhibit the same degree of complexity. It will produce a minimum value within the “plane of complexity vs selection criteria”, thereby corresponding to the non-physical optimum model. The primary distinction between the COMBI algorithm and MIA is the number of layers. According to Anastasakis and Mort (2001), the structure of the multilayer algorithms is comparable to multilayer feedforward neural networks; however, the distinction lies in the number of layers and neurons. These are objectively allocated by the external criterion in compliance with the incompleteness theorem. The principal theory behind GMDH and the GMDH algorithms is framed by four heuristics (Ivakhnenko 1970).

1. Collect a dataset ideally representative of the object sought—for example, rainfall data.
2. Partition the dataset into two subsets; the first is deemed the ‘training’ set by which the polynomial coefficients are determined. The second dataset forms the ‘testing’ set for use with the external criterion allowing separation of the embedded metadata into two divergent categories: helpful or unhelpful. The partitioning is undertaken automatically by the GMDH algorithmic process, requiring no user input. Test data usually comprise 33% of the total dataset, with the algorithm selecting where to draw the data from (Dorn et al. 2012).
3. Produce a set of elementary functions (often quadratic polynomials) delivering increasing complexity through an iterative process where a range of different models are produced.
4. Apply the external criterion for the selection of the optimised model. This procedure is based upon Gödel’s incompleteness theorem, which states “under certain conditions in any language there exist true but unprovable statements” (Uspensky 1994, p. 241). The implication is that for the model most representative of the system to be found, a comparative analysis is required with the external criterion. He et al. (2008) state that the data used within the training set and the external criterion are mutually exclusive. The data that is not used within the training set for estimating the parameters and creating the model from the testing set data is then used by the external criterion for evaluating and selecting the model of the best quality. He et al. (2008) emphasise the significance of optimised cooperation between the external criterion and the division of the dataset; the latter, though, is not an option with GMDH Shell 3. Readers should refer to He et al.’s work for a comprehensive analysis.

The foundational mathematical process that underpins GMDH theory is the Volterra functional series, represented in discrete analogue form as Eq. (1), the Kolmogorov–Gabor polynomial. (Anastasakis and Mort 2001). A nonlinear multivariate high-order polynomial can describe past and present time series (Gilbar 2002). This capability is distinct from a Taylor series that can only describe a specific moment in time. This means the former captures dynamic time representation; the latter is specifically static

1 $$Y=\left(x,t\right)={a}_0+\sum\nolimits_{i=1}^M{a}_i{x}_i+\sum\nolimits_{i=1}^M\sum\nolimits_{j=1}^M{a}_{ij}{x}_i{x}_j+\sum\nolimits_{i=1}^M\sum\nolimits_{j=1}^M\sum\nolimits_{k=1}^M{a}_{ij k}{x}_i{x}_j{x}_k+\dots$$

The model output response is designated *Y*, *x* = (*x*_1_, *x*_2_, *x*_3_, …, *x*_*m*_) the vector of input variables also referred to as regressors where ${x}_m\in {\mathbb{R}}^{m_x}$ , and *a* = (*α*_0_, *α*_1_, *α*_2_, …, *α*_*m*_) the vector of coefficients or weights, and *m* is the number of regressors.

Müller et al. (1998) detail that the GMDH algorithm utilises an inductive approach framed by the self-organisation principle. The inductive approach is unbounded with the regressors randomly shifted and activated allowing for the closest match to the dependent variables to be selected (Madala 1991). Self-organising is, according to Green et al. (1988), a non-parametric process in terms of there being a priori. The idea of a unique model with optimum complexity that can be determined through self-organisation forms the foundation of the inductive approach. GMDH delivers the output through the construction of analytic functions formed by quadratic polynomials in a feedforward network structure. The polynomial coefficients are derived from pairs of regressors through a regression technique based upon the ordinary least squares method (OLS). A cascade of quadratic polynomials, commonly referred to as partial descriptions (PD), resides in each neuron (Madala and Ivakhnenko 1994). The quadratic polynomials being of the form

2 $$y={a}_0+{a}_1{x}_i+{a}_2{x}_j+{a}_3{x}_i^2+{a}_4{x}_j^2+{a}_5{x}_i{x}_j$$

Appendix A illustrates the mathematical functionality of the algorithmic process.

## GMDH limitations

The unsuccessful rainfall modelling that spawned the writing of this paper is not provided here as its inclusion is unnecessary. What is necessary is ascertaining why parametric GMDH was unsuccessful and what can be introduced to facilitate improvement in using GMDH for modelling phenomenon such as rainfall. Limitations encompass a range of factors, including the exclusion of essential regressors initiating noise that impairs model performance (Anastasakis and Mort 2001). From studies undertaken by Green et al. (1988), the problem appears to be caused by collinearity. Even with independent regressors within the input vector, the PDs formed within the first and subsequent iterations were not mutually independent. Green et al. (1988) further emphasised that this results in a selection of regressors being excluded. Overfitting can also be a problem, and when combined with multiple neuron layers, instability delivers poor prediction quality results (Green et al. 1988). Biased estimates of PD coefficients resulting from the application of OLS are an additional shortcoming (Anastasakis and Mort 2001). There is an assumption that the observed output values and estimated output values should be reflected in a Gaussian distribution, meaning that the use of linear regression is justified for PD parameter determination. The reality, though, is this assumption is frequently violated, meaning that OLS is not a suitable method (Anastasakis and Mort 2001). Standard GMDH will also fail with fuzzy input data, meaning a suitably modified GMDH could be appropriate (Anastasakis and Mort 2001). The limitations listed may not seem considerable, but their implications can be significant for the functionality of standard GMDH. For this reason, modified versions of GMDH are explored below. These formats will be shown to deliver improvements over standard GMDH that are worth investigating further, particularly to test their suitability for rainfall modelling.

## Hybridising GMDH

Accurate rainfall prediction is a challenge when using a singular model, so the introduction of a hybrid that combines two models has the potential to deliver performance that exceeds the capabilities of each composing model (Parviz et al. 2021). One example of time series forecasting is hybridising least square support vector machines (LSSVM) with GMDH. In research undertaken by Samsudin et al. (2011) for time series forecasting, the hybrid LSSVM GMDH model delivered more accurate results due to its robust nature and ability to model nonlinear data. Support vector machines (SVM) like GMDH fall under the umbrella of ML. They map the vector of regressors into a designated high-dimensional feature space Z via a nonlinear mapping procedure selected a priori. A hyperplane is constructed within this space that ensures the high generalisation ability of the network (Cortes and Vapnik 1995). The technique of support vector networks was originally developed for limited application in separating training data without errors. The situation often arose where this ideal was not possible, so by extension SVMs were deemed a new class of ML with comparable power and universality to neural networks.

**Fig. 1:**
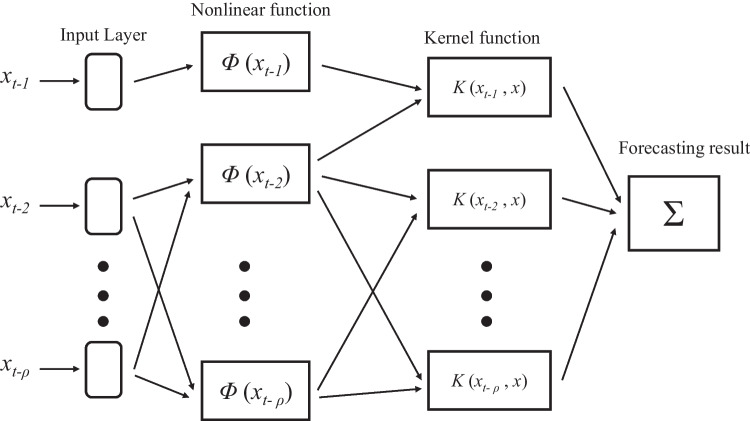
Structure of the support vector machine

Further development introduced the least-squares variant of SVM, designated LSSVM (Cortes and Vapnik 1995). The difference is that the SVM employs equality constraints, whereas the LSSVM uses inequality constraints and implements the system of linear least squares as its loss function (Samsudin et al. 2010a). The LSSVM also offers good convergence combined with high precision, whereas the SVM uses quadratic program solvers that are more challenging to use (Samsudin et al. 2010a). Analogous to GMDH, the LSSVM predictor detailed in Samsudin et al. (2010b) is trained by employing a set of historical time series regressors that deliver a single output as the goal. Appendix B details the mathematical approach of Samsudin et al. (2011). Figure 1 illustrates the structure of the SVM. Most real-world data is nonlinearly separable, meaning the hyperplane is not represented by a straight line. To overcome this problem, Kernel functions are used to allow the transformation of the nonlinear data for linear presentation at higher dimensions (Ampadu 2021). 

The steps taken for hybridisation (Samsudin et al. 2011) are:

1. Separate the normalised data into training and testing sets.
2. Using the GMDH MIA, combinations of all input state variables (*x*_*i*_, *x*_*j*_) are generated, with the totality of independent variables being ${C}_2^M$. The regression polynomial is constructed with an approximation of the output given by equation (A9). The linear vector of coefficients ***A*** for each PD is determined by OLS.
3. The output of each neuron *x*^′^ is assessed against the external criterion with the smallest MSE selected for the formation of a binary input { *x*_1_, *x*_2_, …, *x*_*M*_, *x*^′^} with *M* = *M* + 1, for a neuron within the next hidden layer.
4. In the GMDH output layer from neurons within the hidden layers, these outputs form inputs { *x*_1_, *x*_2_, …, *x*_*M*_, *x*^′^ } for the LSSVM. The minimal MSE from the LSSVM will be selected as the output model.
5. The minimal MSE obtained from the LSSVM for the test data set extracted at each layer during the current iteration is compared against the minimal value from the previous iteration. In the case of an improvement, steps 2 to 4 are repeated. Otherwise, the iterations cease with the knowledge that the network is now complete. Determination of the final layer signifies that only one node with the best performance will realise selection. When this occurs, the remaining nodes with the output layer are discarded, thus delivering the hybrid group least squares support vector machine (GLSSVM) model.

## The Kalman filter and GMDH

In its original form, the Kalman filter (KF) algorithm employs a dynamics model that describes its status and its expected status at the following step, provided the system is linear, with any process disturbance combined with the error of measurement being additive and Gaussian (Pasek and Kaniewski 2021). Most real-world systems that include rainfall data are within the nonlinear category, and in the case of monthly rainfall data, non-Gaussian. To overcome this incompatibility, the extended Kalman filter (EKF) was developed for handling nonlinear functions which are locally approximated with linear equations through Taylor expansion (Pasek and Kaniewski 2021). Monthly rainfall data are highly nonlinear, potentially introducing significant errors in linearisation, as only the first term of the Taylor series is utilised (Pasek and Kaniewski 2021). The EKF also requires the computation of the Jacobian matrix at each time step.

If the initial conditions are poorly known, or there are significant measurement errors, major errors in state vector estimation could potentially lead to divergence of the filter (Kraszewski and Czopik 2017). As a means of overcoming these problems, the unscented Kalman filter (UKF) was developed by Julier and Uhlmann (1997). To implement the UKF, a set of sigma points is chosen with a known mean and covariance. A nonlinear function is applied at each point, yielding a set of transformed points. The transformed point statistics can be calculated to provide an estimate of the nonlinearly transformed mean and covariance (Julier and Uhlmann 2004). In combining the UKF with GMDH, the UKF is utilised for parameter estimation for each GMDH neuron PD (Luzar et al. 2011). GMDH determines the parameters for each PD separately, and it is this procedure that allows the UKF to be utilised (Mrugalski 2013). The main advantage of applying UKF for estimating PD parameters is the generation of an asymptotically stable GMDH model (Mrugalski 2013).

The design of a Kalman filter is normally based upon an analytical model of a dynamic process or system *X*_*k* + 1_ = *F*(*x*_*k*_, *p*), with *x*_*k*_ the unknown state vector for the dynamic process taken at time step *k* (Pan et al. 2020). *F*(.) is the dynamic model of the system parameterised by *p*, a covariance vector.

*y*_*k*_ = *H*(*X*_*k*_, *p*) is the observable variables model belonging to the dynamic system, with *y*_*k*_ the observation vector composed of variables that can be measured (Pan et al. 2020). *H*(.) is the state to measurement matrix, which maps between *x*_*k*_ and *y*_*k*_.

Additive process noise affects the degree of accuracy of *F* which is modelled by *Q*, the covariance matrix. *R****,*** the observation noise covariance matrix, models the uncertainty properties of system observations (Pan et al. 2020).

**Fig. 2:**
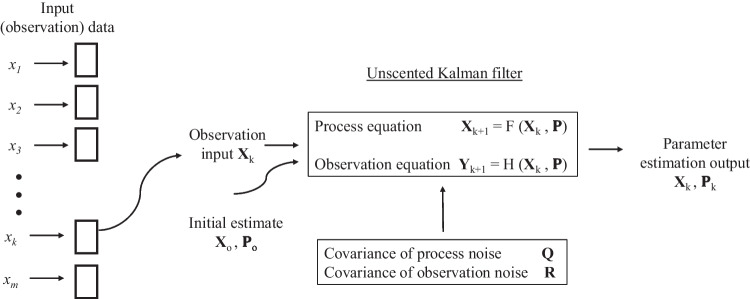
Unscented Kalman filter for determining GMDH neuron parameters

The UKF is initialised by making an estimate of the unknown state variables $\left({\hat{X}}_0\right)$, supplied with various measurements (*y*_*k*_). The UKF state variables $\left({\hat{X}}_0\right)$ are recursively estimated through covariance minimisation ${\hat{P}}_k=\mathit{\operatorname{cov}}\left({x}_k-{\hat{x}}_k\right)$. ${\hat{P}}_k$ is a diagonal matrix with elements representing the uncertainty estimate of ${\hat{x}}_k$ (Pan et al. 2020). Through the implementation of the UKF algorithm, the mean squared estimate errors are minimised with an increase in the convergence rate in addition to a stability improvement against corruption by the noise of the experimental data (Masoumnezhad et al. 2016). For a detailed mathematical explanation of the parameter estimation of GMDH dynamic neurons, refer to Mrugalski (2013). Figure 2 illustrates the integration of the UKF into GMDH for PD parameter estimation. No literature to date has been found where this approach has been applied to rainfall modelling. However, it has been used with success in the identification of various dynamic systems (Luzar et al. 2011) and for the successful design of a robust fault detection system (Mrugalski 2013).

## Signal processing approaches

In research undertaken by Moosavi et al. (2017), hybridising wavelet transform (WT) and wavelet packet transform (WPT) with GMDH (WGMDH and WPGMDH, respectively) delivered improved results over GMDH alone for runoff forecasting. Of these two, the WPT hybrid delivered the best results. Both the WT and WPT improve the performance of the GMDH modelling by decomposing the original dataset into components (Moosavi et al. 2017).

In a study undertaken by Moosavi (2019) predicting rainfall within the context of natural disasters, GMDH was hybridised with ensemble empirical mode decomposition (EEMD), and again with WT, and WPT forming WTGMDH and WPTGMDH, respectively. In all three cases, the rainfall modelling with GMDH improved once hybridised with these signal processing approaches. The WPTGMDH performed best, followed by EEMD-GMDH, then WTGMDH. All these hybrids outperformed the standard GMDH without signal processing assistance. In further research by Moosavi et al. (2021), the combination of GMDH with the same three separate signal processing approaches—EEMD, WT, and WPT—improved the performance of GMDH for groundwater level forecasting in all three cases.

**Fig. 3:**
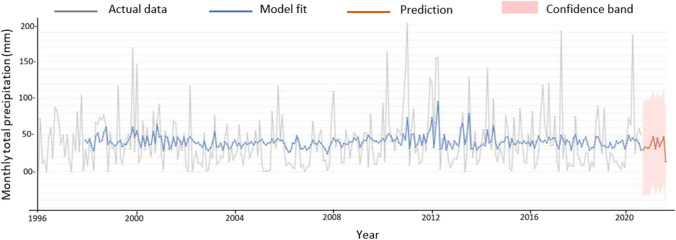
GMDH model of monthly rainfall in Forbes NSW from 1995 to 2021

Given these findings, it seems prudent to investigate further the potential for improving the modelling of monthly rainfall data with GMDH by pre-processing the data with these signal processing approaches. An example of the monthly rainfall modelling undertaken during 2020–2021 using GMDH is illustrated in Fig. 3, covering the town of Forbes from 1995 to 2021.

In Fig. 3, the BoM (2020) data profile is formed by the grey graph, while the blue graph illustrates the GMDH model, with the red being the prediction over 12 months into the future. The coefficient of determination returned a value of 0.246. All rainfall modelling undertaken returned similar unsatisfactory results. There is no clear trend depicted within the actual data, nor can past events be a reliable means of predicting future events. The focus of this empirical assessment is to consider whether pre-processing the rainfall data might improve the modelling outcome, thereby providing greater confidence in the rainfall prediction.

## Ensemble empirical mode decomposition (EEMD)

Empirical mode decomposition (EMD) was introduced by Huang et al. (1996). It is a method that is applicable for both nonlinear and non-stationary time series with Srikanthan et al. (2011) noting that the EMD method makes no assumption about the form of the time series prior to analysis. The EMD can adaptively decompose any complex dataset into a series of intrinsic mode functions (IMF) (Huang et al. 1996). The IMFs are independent, and in combination with a residual can effectively be summed together to reform the original series (Srikanthan et al. 2011). EMD does, however, experience mode mixing, which occurs when at a given frequency a fluctuation may separate across two IMFs. Specifically, mode mixing often occurs from intermittent signals (Wu and Huang 2009). The EMD algorithm employs a data-driven adaptive iterative approach meaning that there is difficulty in avoiding mode mixing without subjectively contemplating the likely form of any signal for extraction before undertaking analysis (Srikanthan et al. 2011).

**Fig. 4:**
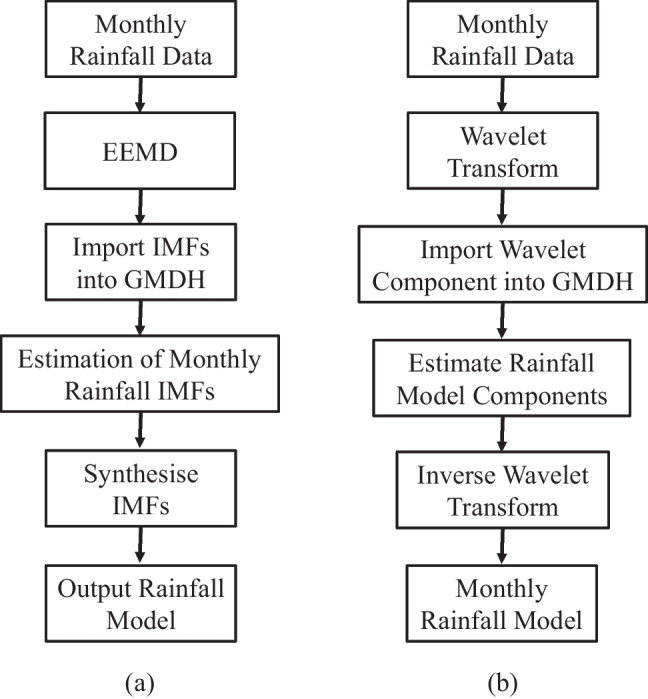
Flowchart for the **a** hybridised EEMD-GMDH algorithm and **b** hybridised WT-GMDH algorithm

A noise-assisted data analysis (NADA) is proposed to overcome the mode mixing problems. The ensemble EMD (EEMD) provides the definition of the true IMF components “as the mean of an ensemble of trials, each consisting of the signal plus white noise of finite amplitude” (Wu and Huang 2009, p. 2). Looking more closely at the EMD method and then introducing white noise for the EEMD approach, refer to Appendix C for details on the process of decomposing the signal into IMFs. In utilising the EEMD, the decomposition effect is that the augmented white noise series cancel out in the final mean of the corresponding IMFs (Wu and Huang 2009). The mean IMFs are positioned within the natural dyadic filter windows, thereby significantly reducing the potential of mode mixing while preserving the dyadic property (Wu and Huang 2009). The IMFs are more effective in isolating physical processes across a range of time scales owing to mutual orthogonality (Molla et al. 2006). Applying EEMD into the GMDH rainfall modelling is based upon the historical rainfall data signal being decomposed into a set of IMFs with a residual prior to processing by GMDH. The process that GMDH takes is through implementing supervised learning, with IMFs being supplied as input data. That process and the formation of a function to represent the data, enabling the capacity to predict future rainfall events, should in theory be improved. Figure 4a illustrates a flowchart of the EEMD-GMDH hybrid.

## Wavelet transform (WT)

Like EEMD, the WT provides a mechanism for signal decomposition. Wavelets are functions that meet a set of mathematical conditions with the ability to represent data and other functions (Graps 1995). They can analyse non-stationary time series across a range of frequencies (Daubechies 1990). By decomposing a time series into time-frequency space, determination of the dominant modes of variability can be achieved, and their time invariance. Wavelet transforms are available in both continuous and discrete forms and both types have been used in the modelling of hydrometeorological studies (Torrence and Compo 1998). The fundamental idea behind WT is their ability to perform signal analysis at different resolutions, which precipitates their most important feature, that of multiresolution decomposition (Lubis et al. 2017). Özger et al. (2012) employed the continuous WT (CWT) in their study of drought forecasting, while Alfa et al. (2019) utilised the discrete WT (DWT) in a hybrid with GMDH for their drought forecasting study. Moosavi et al. (2021) employed the DWT as a hybrid with GMDH in their research on groundwater level modelling. Appendix D provides a brief mathematical overview with reference to Addison (2018) and Lambers (2006).

It is important to recognise that the integral defining Wf(*m*, *n*) exists across an unbounded interval, but effectively it exists across a finite interval provided the mother wavelet has compact support, hence a numerical approximation is easy. Moosavi et al. (2021) detailed the importance of selecting the best WT structure, choosing the mother wavelet and the most effective level of decomposition. The decomposed data is then supplied to GMDH for processing with the optimum model delivered. Figure 4b illustrates a flowchart featuring the integration of the WT with GMDH.

## Wavelet packet transform (WPT)

The WPT improves the WT by raising the degree of resolution of high-frequency signals precipitating an enhanced high-frequency time-frequency localisation effect (Yan et al. 2021). The WPT prevents the loss of time-frequency information, the signal placed within a domain where simultaneous analysis in both time and frequency can occur (Wickerhauser 1991; Gokhale and Khanduja 2010). Wavelet packet decomposition (WPD) involves passing the signal through a greater number of filters compared to the WT (Gokhale and Khanduja 2010). In general terms, a WPT is a square-integrable function with a mean of zero and compact support, across both time and frequency (Zha et al. 2008). A brief mathematical outline by Zha et al. (2008) is provided. In describing wavelet packets by a collection of functions ${\left\{{\varphi}_k\right\}}_k^{\infty }$ is obtained from:

3 $${\varphi}_{2k}(x)=\sqrt{2}\sum\nolimits_n{h}_k(n){\varphi}_k\left(2x-n\right)$$

4 $${\varphi}_{2k+1}(x)=\sqrt{2}\sum_n{g}_k(n){\varphi}_k\left(2x-n\right)$$

noting that the discrete filter *h*_*k*_(*n*) and *g*_*k*_(*n*) are quadrature mirror filters (p. 405). The function *φ*_0_(*x*) is identifiable with the scaling function, and *φ*_1_(*x*) with the mother wavelet. The wavelet packet basis, due to its orthonormality and completeness, guarantees retention of the original signal information. The inverse transform of the wavelet packets can be expressed as:

5 $${\varphi}_k(x)=\sum\nolimits_n\left[{h}_k(n){\varphi}_{2k}\left(x-2n\right)+{g}_k(n){\varphi}_{2k+1}\left(x-2n\right)\right]$$

**Fig. 5:**
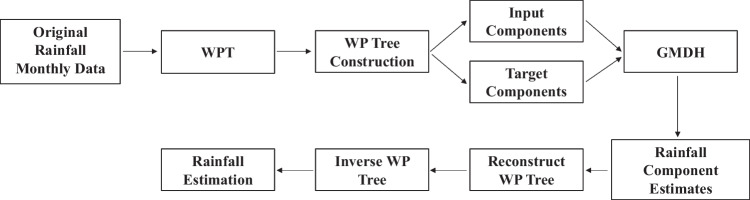
Flowchart for hybridised WPT with GMDH

The potentially significant component of the WPT specifically within this paper and the context of rainfall modelling is its ability to recursively decompose high-frequency signal components (Zha et al. 2008). The WPT constructs a tree formation multiband extension of the WT. This facilitates the ability of the WPT to recursively divide the entire frequency band for noise detection and removal. By sifting the signal of components that can be classed as noise within the context of isolating the intense rainfall events, it is hypothesised that the capacity of the hybridised WPTGMDH to produce models with an improved coefficient of determination is possible. Such could potentially highlight trends within the rainfall data with greater clarity and prediction significance. Figure 5 illustrates the hybrid WPTGMDH modelling process.

## Improved GMDH algorithms

MIA and COMBI algorithms share the same approach to determining the PD coefficients, that of OLS. This regression approach can be subject to the production of coefficients with biased estimates, which is detrimental to model accuracy. Structurally, the MIA is superior to the COMBI algorithm by virtue of possessing multiple layers of neurons which make processing time more expedient. One method for improving the performance of the MIA algorithm can be found within the neuron pruning process (Buryan 2013). In the MIA, the pruning of neurons based upon the external criterion can result in the loss of neurons that may have been useful. Synthesising the neuron outputs within a given layer promotes ill-performing regression within the next layer (Buryan 2013). The network’s complexity can exceed levels desirable owing to the quadratic polynomials that form the PDs when the vector of regressors is highly nonlinear (Buryan and Onwubolu 2011). To overcome these shortcomings, the enhanced MIA-GMDH (eGMDH) or eMIA-GMDH algorithm is proposed, detailed by Buryan and Onwubolu (2011) from work undertaken by Buryan (2006).

## Enhanced MIA-GMDH

The improvements encompass the following (Buryan and Onwubolu 2011):

1. Within the original GMDH, equation (A2) *n* = 2 for all layers, with eGMDH application applies only within layer one. All subsequent layers can utilise different values of *n*.
2. Utilising OLS for PD coefficient determination is not limited to quadratic polynomials, instead including a further seven types which modify Eq. (2).

a Harmonic based on the cosine function
b Radical
c Inverse polynomial
d Natural logarithm
e Exponential
f Arc tangent
g Rounded polynomial presenting integer coefficients

2. The pruning of layers is semi-randomised. Upon selecting a subset of the best-performing neurons in each layer, the balance is selected at random.
3. Implementation of coefficient rounding and thresholding as a means of stabilising the regression process by rejecting coefficients that lay either above or below pre-set thresholds.
4. Neuron inputs are not binary limited.

From experiments undertaken by Buryan (2013), the enhanced MIA-GMDH delivered improved results when compared to the standard MIA-GMDH algorithm for both Mackay-Glass time series predictions and JPY/USD exchange rate predictions for both daily and monthly timeframes. In a study undertaken by Unwubolu et al. (2007) covering weather forecasting in Fiji, the eGMDH delivered improved results when compared to a PNN and an enhanced PNN (ePNN) for temperature modelling, but it was less successful for rainfall modelling against the alternatives. The study concluded with an unclear finding as to why this was the case.

## Enhanced GMDH using modified Levenberg-Marquardt method

Pournasir et al. (2013) proposed an enhanced GMDH algorithm utilising a modified Levenberg-Marquardt (LM) method, which incorporated the use of singular value decomposition (SVD) for the first time as an improved means for the first guess. It is noted that the process of combining the SVD output into LM for the initial guess was not detailed in their paper. The LM algorithm facilitates swift convergence for solving nonlinear systems and singularity problems (Pournasir et al. 2013). The experimental outcome illustrates that the enhanced GMDH using the LM algorithm outperformed standard GMDH in delivering results with high inventory control accuracy (Pournasir et al. 2013). In fitting a parameterised mathematical model to a set of data points (within the focal context of the initiator of this report—rainfall modelling) through minimisation of an objective function, which within the context of GMDH is the selection criterion—the MSE—, the problem of least squares arises. Minimising the objective with respect to the parameters may be possible with speed, provided the solution fits a linear matrix equation (Gavin 2020). However, if this is not the case and the fit function is not linear within its parameters, then the least squares problem needs an iterative algorithmic solution (Gavin 2020). The algorithmic process involves reducing the sum of the squares of the errors between the function representative of the model, and the data points through a sequence of carefully selected model parameter updates (Gavin 2020). The LM algorithm merges two numerical minimisation procedures: the gradient descent (GD) method and the Gauss-Newton (GN) method. In the former case, by updating the parameters in the direction of steepest descent, the sum of the squared errors is reduced. For the latter, the assumption is made that the least squares function is locally quadratic in the parameters, and the sum of the squared errors is reduced, thereby allowing the minimum of the quadratic to be found (Gavin 2020). When the parameters are far removed from their optimal value, the LM behaves akin to the GD method, and when the parameters are close to their optimal target, the LM acts like the GN method (Gavin 2020). Appendix E details mathematically the integration of LM with GMDH.

Integrating LM with GMDH is presented in pseudocode (Transtrum et al. 2011; Mulashani et al. 2021).

1. Input rainfall data into GMDH
2. Determine the initial information for the LM-GMDH structure—initial point *x*_0_, damping parameter *λ*, *λ*_↑_, *λ*_↓_ for damping term adjustment
3. Estimate the parameter weights
4. Evaluate the Jacobian at the initial parameter value and the residuals
5. Calculate the metric Γ = *J*^T^*J* + *λI*, the objective function $f(x)=\frac{1}{2}{\left|r(x)\right|}^2$, and ∇*f*(*x*) = *J*^T^*r*
6. Calculate the new residuals *r*_new_ at the location provided by *x*_new_ = *x* − Γ^−1^ ∇ *f*(*x*),then calculate the objective function at the new location $f{(x)}_{\mathrm{new}}=\frac{1}{2}{\left|r{(x)}_{\mathrm{new}}\right|}^2$
7. If *f*(*x*)_new_ < *f*(*x*), accept the step, assign *x*_new_ to *x* and set *r* = *r*_new_ then assign $\lambda =\frac{\lambda }{\lambda_{\mathrm{down}}}$ else reject the step, retain the old parameter guess *x* and residuals, setting *λ* = *λλ*_up_
8. Convergence assessment. If there is convergence, return *x* as the best fitting parameter. If there is no convergence but the step was accepted, calculate the Jacobian at the new parameter values and return to step 5.
9. Assess the PDs within the current layer against the external criterion
10. Retain the best-performing neurons
11. If the current layer is the final layer, terminate, else return to step 2.

## Fuzzy GMDH

In non-fuzzy or standard GMDH, the residuals between the observed output values and estimated output values are assumed to be Gaussian, thereby allowing parameter estimation through linear regression (Anastasakis and Mort 2001). This assumption though does not always hold, meaning OLS is not appropriate (Anastasakis and Mort 2001). It follows that real-world systems adhere to Zadeh’s (1980) principle of incompatibility, thus the modelling procedure and theory that is more appropriate is fuzzy (Anastasakis and Mort 2001). In dealing with the fuzzy phenomenon and fuzzy data, Tanaka et al. (1989) suggest utilising possibility measures to describe the fuzzy system equation. Variations between observed and model values are normally categorised as measurement errors within a standard regression model (Tanaka et al. 1989). Within the fuzzy context, the system parameters are considered responsible; correspondingly, the deviations are reflected in possibilistic linear systems (Tanaka et al. 1989). In fuzzy GMDH (FGMDH), the architecture remains unchanged, but the PDs contain fuzzy parameters, which, to be found, require possibilistic linear regression (Anastasakis and Mort 2001). A mathematical description as detailed by Hayashi and Tanaka (1990); Fuzzy number *M*, *μ*_*M*_ : *ℝ* → [0, 1], satisfies:

6 $${M}_{\lambda }=\left\{x:{\mu}_M(x)\ge \lambda \right\}\to \mathrm{closed}\ \mathrm{interval}$$

7 $$\exists x\ \mathrm{such}\ \mathrm{that}\ {\mu}_M(x)=1$$

8 $${\mu}_M\left(\lambda {x}_1+\left(1-\lambda \right){x}_2\right)\ge {\mu}_M\left({x}_1\right)\bigwedge \kern0.5em {\mu}_M\left({x}_2\right)\ \mathrm{with}\ \lambda \in \left[0,1\right]$$

An alternative to linear or quadratic polynomials that form the PDs is orthogonal polynomials (Zaychenko and Zaychenko 2019). Their orthogonality precipitates faster coefficient determination than non-orthogonal polynomials, and the coefficients are not dependent upon the initial polynomial degree (Zaychenko and Zaychenko 2019). Chebyshev’s polynomial approximation is a form of regression that minimise autocorrelation between the model response and the sampling locations (Nakajima 2006). Chebyshev orthogonal polynomials are especially well suited to equally spaced sample points (Nakajima 2006), which is the case with the BoM rainfall data. A mathematical representation of the general case of Chebyshev’s orthogonal polynomials can be found in Zaychenko and Zayets (2001). PDs featuring trigonometric polynomials are also an option with FGMDH with a mathematical outline provided within Zaychenko and Zaychenko’s (2019) paper. The advantage that FGMDH offers over standard MIA-GMDH is the zero requirements to use OLS for determining the PD parameters. Fuzzy GMDH can be used with both crisp and fuzzy regressors, which extend its application for potential rainfall modelling. Zaychenko and Zaychenko (2019) reported high accuracy of results when modelling with FGMDH for forecasting financial processes. Shi et al. (2020) found that FGMDH identified regional economic bottlenecks within China with high recognition accuracy compared to standard GMDH. Panchal et al. (2014), in their study of rainfall-runoff modelling using fuzzy logic, returned a coefficient of determination of 0.988. They did not combine it with GMDH, but the illustration shows the gains that can be achieved with the fuzzy approach. No papers have been located to date that uses FGMDH for rainfall modelling.

## Neuro-fuzzy GMDH

Neuro-fuzzy is derived from the application of Gaussian radial basis functions (GRBF) as PDs (Anastasakis and Mort 2001). Radial basis functions (RBF) are univariate functions that provide a mechanism to approximate multivariate functions through linear combinations of terms (Buhmann 2010). In considering the GRBF as fuzzy production rules, Mamdani (1976) explored a fuzzy reasoning rule: Let *x*_1_ = *A*_*k*1_ and *x*_2_ = *A*_*k*2_ then output *y* = *w*_*k*_. Letting the Gaussian membership function *A*_*kj*_ of the *k*th fuzzy rule (*k* = 1, …, 4) in the domain of the *j*th input variables *x*_*j*_(*j* = 1, 2) be defined as:

9 $${A}_{kj}\left({x}_j\right)=\exp \left\{-\frac{{\left({x}_j-{a}_{kj}\right)}^2}{b_{kj}}\right\}$$

with parameters *a*_*kj*_ and *b*_*kj*_ being given for each rule. Let *w*_*k*_ be a real number from the conclusion of the *k*th rule suggesting model output *y*. The degree of compatibility from the proposition is:

10 $${\mu}_k=\prod_{j-1}^2{A}_{kj}\left({x}_j\right)$$

with the model output defined

11 $$y=\sum\nolimits_{k=1}^4{\mu}_k{w}_k$$

The GRBF ↔*y* → is a neural network composed of three layers (Poggio and Girosi 1990). The neuro-fuzzy GMDH (NFGMDH) is formed through the implementation of this fuzzy reasoning model as the PDs (Mucciardi 1972, as cited in Nagasaka et al. 1995). NFGMDH follows the same paradigm as standard GMDH. Within the context of the GRBF as PDs, the inputs from the *β*th model and *ρ*th layer form the output variables of the (*β* − 1)th and *β*th model within the (*ρ* − 1)th layer (Nagasaka et al. 1995; Najafzadeh 2015). The mathematical expression for ascertaining *y*^*ρβ*^ is illustrated as:

12 $${y}^{\rho \beta}=f\left({y}^{\rho -1,\beta -1},{y}^{\rho -1,\beta}\right)=\sum\nolimits_{k=1}^K{\mu}_k^{\rho \beta}{w}_k^{\rho \beta}$$

13 $${\mu}_k^{\rho \beta}=\exp \left\{-\frac{{\left({y}^{\rho -1,\beta -1}-{a}_{k,1}^{\rho \beta}\right)}^2}{b_{k,1}^{\rho \beta}}-\frac{{\left({y}^{\rho -1,\beta }-{a}_{k,2}^{\rho \beta}\right)}^2}{b_{k,2}^{\rho \beta}}\right\}$$

with ${\mu}_k^{\rho \beta}$and ${w}_k^{\rho \beta}$ as the *k*th Gaussian function with its associated weight parameter respectively. Additionally, ${a}_k^{\rho \beta}$ and ${b}_k^{\rho \beta}$ are the Gaussian parameters that feature for the *i*th input variable supplied by the *β*th model and *ρ*th layer (Nagasaka et al. 1995; Najafzadeh 2015). The complete model output is illustrated with:

14 $$y=\frac1M\sum\nolimits_{m=1}^My^{\rho\beta}$$

through each iteration of the network model construction, the error parameter is:

15 $$E=\frac{{\left({y}^{\ast }-y\right)}^2}{2}$$

with *y*^∗^ the predicted output value (Nagasaka et al. 1995; Najafzadeh 2015).

In terms of successful outcomes, Nagasaka et al. (1995) reported from their research in utilising NFGMDH for modelling grinding characteristics with GRBF PDs that it delivered an improved result when compared with standard GMDH. Yousefpour and Ahmadpour (2011), from their research into air pollution predictions with NFGMDH, found improved results when compared to those obtained from a multilayer perceptron (MLP) neural network. Miyagishi et al. (2010) found an improvement when compared to the Kalman filter for temperature prediction when the seasonal change was moderate. No papers were located where NFGMDH was used to model rainfall data.

## Exogenous data

Exogenous data is the additional data that is not required to run a model but provides an increase in modelling accuracy. Such data was used in the research study undertaken by Moosavi (2019), investigating the impact of rainfall on natural disasters. GMDH was utilised with three signal processing approaches EEMD, WT, and WPT, in addition to exogenous data. Two distinct datasets were used to predict rainfall, one without exogenous data and one with exogenous data. The exogenous data were evaporation, minimum and maximum temperature, and humidity. All data was on a monthly timescale. Modelling rainfall with 1-to-4-month forecasts using just GMDH delivered poor performance. With the inclusion of the exogenous data, the GMDH modelling was an improvement on the modelling that excluded the exogenous data but overall was still poor. Hybridising GMDH with each of the signal processing techniques in turn improved the rainfall modelling, which further improved when the exogenous data were included. Exogenous data were used by Mendoza et al. (2020) for their research study into rainfall in Ecuador. The modelling was undertaken using a dynamic harmonic regression framework where global climate signals formed the exogenous data. Their results were favourable, illustrating greater reliability and robustness against limitations within the data. In a study undertaken by Sarveswararao and Ravi (2020) covering ATM cash demand forecasting, GMDH was supplied with a dummy exogenous variable in the form of the day of the week. Supplied with this information, the GMDH modelling delivered an improvement in the symmetric mean absolute percentage error (SMAPE) when compared to the data modelling without inclusion of the dummy exogenous variable. From the evidence presented, the GMDH paradigm is accepting of the inclusion of exogenous data, which improves the success of the model.

## Discussion

As a mechanism for enhancing the usability and possibly the capacity of standard GMDH to model rainfall data, this empirical study considers alternative platforms for further investigation. Gaining an appreciation and deeper understanding of the GMDH algorithm could allow for greater modelling success. Luzar and Witczak (2014) utilised MATLAB for implementing the algorithmic idea of GMDH. Advantages of this approach include state vector dimensions, choice of multiple selection criteria, and stopping criteria. Onwubolu (2014) details the implementation of GMDH in C programming language, and Onwubolu (2016) describes the implementation of the GMDH paradigm into MATLAB. Dag and Yozgatligil (2016) investigated short-term forecasting with a GMDH R-package running within the R-workspace. This option allows the implementation of different transfer functions precipitating the selection that delivers the smallest prediction MSE. The Hilbert-Huang transform (HHT) method, although not specifically within the scope of this paper, is worth mentioning as it is a combination of EMD and Hilbert spectral analysis (HSA) (Huang and Shen 2014). The HHT is potentially suitable for analysing nonlinear and non-stationary data (Huang and Shen 2014). Bowman and Lees (2013) discuss the HHT package available for the R programming language. As GMDH can also be implemented with the R platform, the potential for pre-processing the rainfall data with HHT cannot be denied. This paper suggests that this combination would be a viable option for further investigation.

## Conclusion

The GMDH hierarchical structure of the COMBI and MIA algorithms has been discussed in this paper with an investigation of state variable distribution, their classification, and the synthesis of PDs. The limitations of OLS in determining PD coefficients, the inherent potential for biased estimates, the significance of fuzzy input data, and the integration of Gödel’s incompleteness theorem point to the requirement of an external criterion. The methods for modelling improvement covered hybridising with LSSVM, which was shown to be successful for time series forecasting, although modelling of rainfall data appears not to have yet taken place. In implementing LSSVM, normalised data is a requirement, which is distinct from how data is provided to standard GMDH. This provides an opportunity to test the application of normalised data for both standard GMDH and hybridised form and allows for further research in utilising LSSVM with GMDH for rainfall modelling and forecasting. The integration of Kalman filters into the GMDH paradigm for the forming of PD parameters was detailed with success in this application in the modelling of dynamic systems and fault detection. This pairing has not yet been applied to rainfall modelling and forecasting, leaving the opportunity for further research in this area. By hybridising GMDH with three signal processing techniques; EEMD, WT, and WPT, all delivered an improvement for rainfall prediction within the context of natural disasters, with WPTGMDH delivering the largest improvement. It would be interesting to see the outcome of further rainfall modelling using these signal processing techniques within the Australian context, particularly if it covered the same six LGAs in the first author’s original study.

Enhanced MIA-GMDH received exposure to rainfall modelling but delivered results that were deemed unsatisfactory, quite distinct from the success in the temperature modelling. This paper recommends that this would be an ideal area for further research encompassing rainfall modelling through hybridisation with one or more of the applications already discussed. The hybridising of the LM algorithm with GMDH for determining the PD parameters was very successful for the high degree of accuracy associated with inventory control. This pairing has not yet been found to have received the application of rainfall modelling, providing an opportunity for further research in this area. It would be interesting to see what benefit the modelling has for rainfall forecasting when the PD parameters are not within the confines of OLS. Fuzzy GMDH finds favour when modelling real-world systems, given their inherently fuzzy nature resulting in adherence to Zadeh’s principle of incompatibility. The PD parameters within FGMDH require determination by possibilistic linear regression or they take an alternative form of orthogonal polynomials. FGMDH can see the application of modelling with both crisp and fuzzy input vector regressors thereby widening the base of the application. Fuzzy logic as distinct from FGMDH was used with great success in Panchal et al. (2014) study of rainfall-runoff modelling, returning a coefficient of determination of 0.988. This paper highlights the potential for further research into rainfall modelling and forecasting with the application of FGMDH. NFGMDH, which utilises GRBFs as PDs, found success in terms of an improvement in previous studies that modelled grinding characteristics and for air pollution predictions when compared to standard GMDH and an MLP. No studies have been found where NFGMDH has been applied to rainfall modelling, but the opportunity exists for further research in this area. The inclusion of exogenous data improved the modelling for each of the cited studies. This paper considers it a worthwhile proposition to include exogenous data in all the modelling avenues discussed, as the evidence suggests an improvement in the model will be achieved.

### Supplementary information

ESM 1 (DOCX 195 kb)

## Data Availability

Not applicable.
