# Supplementary material for: Review of the limitations and potential empirical improvements of the parametric group method of data handling for rainfall modelling
Source: Environ Sci Pollut Res Int. 2022 Oct 10;30(44):98907–21. doi: 10.1007/s11356-022-23194-3 (PMC10533576; doi:10.1007/s11356-022-23194-3)
Supplement: Supplementary file 1 — (DOCX 195 kb) [file 11356_2022_23194_MOESM1_ESM.docx]

# Appendices

## Appendix A

In defining a nonlinear system encompassing rainfall data with the multilayer algorithm considered first:

$y =f(x_{1}, x_{2},\ldots, x_{n})$ (A1)

where $x_{1}, x_{2},\ldots, x_{n}$ and $y$ form the state system variables. The input variables are termed the learning set describes Jiřina and Jiřina jr (2013), being normally divided into “two disjoint subsets”, the first being the training set, the second being the validation set (p. 455). During the learning process, the PD coefficients are formed from elements within the training set, the elements from the validation set expose the magnitude of the error allowing for PD retention or disposal. The function $f$ links these variables through the relationship defined by the Kolmogorov – Gabor polynomial. Nikolaev and Iba (2003) highlight the fact that the Weierstrass theorem illustrates that these polynomials are a format that is universal for “non-linear function modelling”. They can represent approximately any continuous function defined on a compact set to a degree of precision that is arbitrary, within the context of an average squared residual (ASR), provided the number of terms is sufficient. The series is not infinite in practice, instead truncated due to design decisions (p. 1528). Each GMDH active neuron houses a PD, accepting two inputs then producing a single output. Within the first network layer, Ivakhnenko (1970) and Dorn et al. (2012) define the neuron number as: -

$m= C_{2}^{n}= \frac{n^{2}-n}{2}$ (A2)

where $m$ is the neuron (PD quantity) within the first layer, and $n$ is the number of inputs selected for that layer. From each neuron (PD), the output takes the form: -

$$h_{11}=f_{11}(x_{1}, x_{2})$$

$$h_{12}=f_{12}\left( x_{1},x_{3} \right)$$

$h_{1m}=f_{1m}(x_{n-1},x_{n})$ (A3)

Training of the first layer is undertaken with a comparative analysis of neuron outputs against the external criterion. The external criterion is formed from a vector of regressors not used within the training data set, i.e., a selection criterion. Removal of neurons delivering the least favourable results takes place thereby only retaining those neurons (PD) that best fit the criterion. The selected neurons form a subset of the original set of neurons within the first layer.

$\left[ \hat{h}_{11},\hat{h}_{12},\ldots,\hat{h}_{1\hat{m}} \right]\subset[h_{11},h_{12},\ldots,h_{1m}]$;

$\hat{[f}_{11},\hat{f}_{12},\ldots,\hat{f}_{1\hat{m}}]\subset[f_{11},f_{12},\ldots,f_{1m}]$ (A4)

noting that $\hat{m}<m$ where $\hat{m}$ are the selected neurons from the first layer.

A second layer of neurons is now formed designated as $p$ with the $\hat{m}$ selected neurons from the first layer sampled in pairs forming the new layer $p=C_{2}^{\hat{m}}$. In essentially the same format as eq. (A3), the outputs from $p$ can be defined as: -

$$h_{21}=f_{21}(\hat{h}_{11},\hat{h}_{12})$$

$$h_{22}=f_{22}(\hat{h}_{11},\hat{h}_{13})$$

$h_{2p}=f_{2p}(\hat{h}_{1\left( \hat{m}-1 \right)},\hat{h}_{1\hat{m}})$ (A5)

Neuron selection and the addition of new layers continues until the optimum model is reached, with the criterion of regularity being based upon the mean square error (MSE) with Figure 1A illustrating. The minimal value of the MSE corresponds with the optimal model complexity.


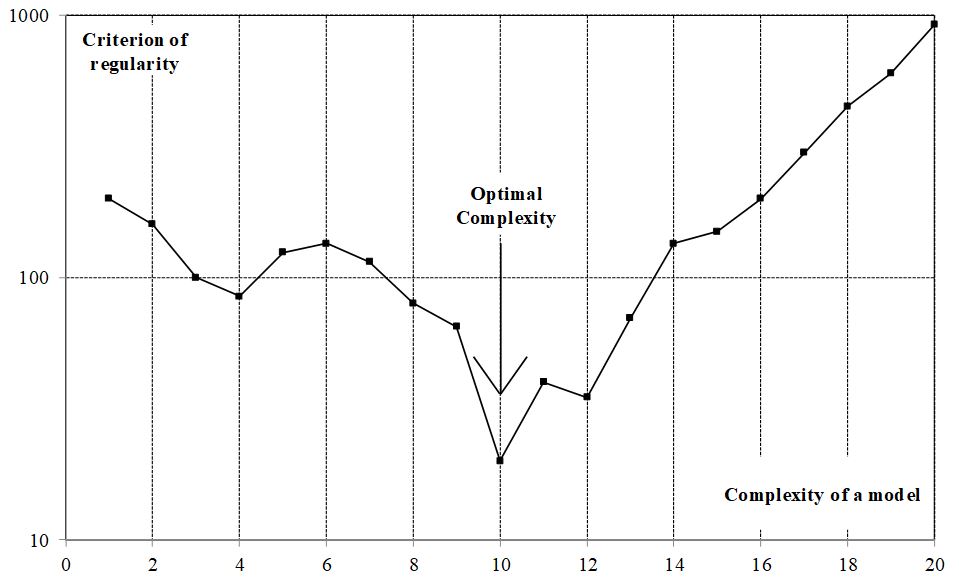


**Fig 1A**. Selection of the optimised model

The poorest performing neurons are removed so only those that fit the criterion are retained, with the best performing often referred to as the Ivakhnenko polynomial. The selection process tests the training data set with the test data against the external criterion. The output from the MSE for each data selection as defined by Ivakhnenko (1970) and Dorn et al. (2012) is: -

MSE$\left( h_{ln} \right)= \frac{1}{s}\sum_{t=1}^{s} {(h_{ln}\left( t \right)-y\left( t \right))}^{2}$ (A6)

where $h_{ln}(t)$ is the output from the partial description within neuron $n$ in the layer $l$ at time $t$, $s$ refers to the number of elements within the sample space of input variables and $y(t)$ is the network model output that is desired. The GMDH training procedure incorporates the addition of layers, the determination of coefficients associated with each partial description, and the elimination of neurons that produce the poorest results. Outputs that are retained from one layer form the basis of inputs for next layer. A halt to the training process occurs when the number of neurons within the current layer falls to one following application of the external complement, or when a subsequent layer does not improve the overall network performance. Should that occur, that last layer is then removed. Preservation of the neuron from the previous layer that delivered the best performance is retained while all other neurons within that layer are discarded. Pham and Liu (1994) describe the trimming process, that of removing all neurons from all layers that do not contribute to the final output. Figure 2A illustrates MIA algorithm architecture. The bold lines illustrate the active connections between the inputs and neurons, and between neurons and neurons. All selected neurons also appear in bold, having met the requirements of the selection criterion.


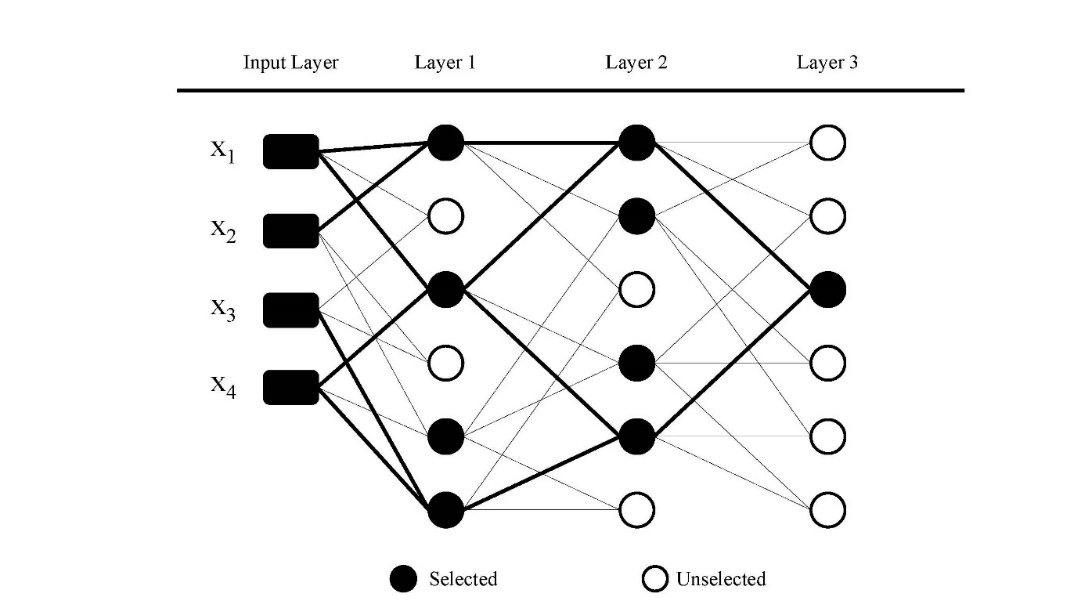


**Fig 2A**. GMDH Multilayer Algorithm

Each neuron within each layer accepts two inputs, producing a single output as described by Ivakhnenko (1970) and Dorn et al. (2012). The inputs are of the form $x_{i}$and $x_{j}$ which are supplied to a PD with output $y$. The linear coefficients are $(a_{0},\ldots,a_{5})$. The PD where $\left( x_{i},x_{j} \right)\subset\mathbf{x}$ ; $i \neq j$ (Madala & Ivakhnenko, 1994) is of the form of eq. (2)

or represented in matrix format as

$h= \mathbf{xa}$ (A7)

where $\mathbf{x}$ is the row vector of pre-processed inputs $x_{i}$ and $x_{j}$ for one PD, conveyed by

$\mathbf{x}=(1, x_{i}, x_{j}, x_{i}^{2},x_{j}^{2},\left( x_{i},x_{j} \right))$ Pham and Liu (1994), and $\mathbf{a}$ is a column vector containing elements of coefficients that have not yet been determined $\mathbf{a}=(a_{0},\ldots,a_{5})^{T}$. For time series rainfall forecasting, the assumption is made that there are $s$ elements within the sample space selected by the algorithm:

$$h\left( 1 \right)=f(x_{1}\left( 1 \right), x_{2}\left( 1 \right),\ldots, x_{n}\left( 1 \right))$$

$h\left( 2 \right)=f(x_{1}\left( 2 \right), x_{2}\left( 2 \right),\ldots, x_{n}\left( 2 \right))$ (A8)

$$h\left( s \right)=f(x_{1}\left( s \right), x_{2}\left( s \right),\ldots, x_{n}\left( s \right))$$

A time series PD will deliver an output at time $t$ supplied by two inputs $x_{i}(t)$ and $x_{j}(t)$

$h(t)= a_{0}+a_{1}x_{i}(t)+ a_{2}x_{j}(t)+a_{3}x_{i}^{2}(t)+a_{4}x_{j}^{2}(t)+a_{5}x_{i}(t)x_{j}(t)$ (A9)

where $t=1, 2,\ldots, s.$

If we now let $h\left( t \right)=y(t)$, (A9) can be portrayed as

$\boldsymbol{y}=\mathbf{Xa}$ (A10)

where

$\mathbf{X=}$ $\left( \begin{matrix} 1 & x_{i}(1) & x_{j}(1) & x_{i}^{2}(1) & x_{j}^{2}(1) & x_{i}(1)x_{j}(1) \\ 1 & x_{i}(2) & x_{j}(2) & x_{i}^{2}(2) & x_{j}^{2}(2) & x_{i}(2)x_{j}(2) \\ \ldots& \ldots& \ldots& \ldots& \ldots& \ldots\\ 1 & x_{i}(s) & x_{j}(s) & x_{i}^{2}(s) & x_{j}^{2}(s) & x_{i}(s)x_{j}(s) \end{matrix} \right)$ (A11)

and

$\boldsymbol{y}$ $=({y\left( 1 \right) y\left( 2 \right) \ldots y\left( s \right))}^{T}$ (A12)

From (13), the matrix of coefficients for the partial descriptions are calculated using the LSM: -

$\mathbf{a}={(\boldsymbol{X}^{\boldsymbol{T}}\boldsymbol{X})}^{-1}\boldsymbol{X}^{T}\boldsymbol{y}$ (A13)

Green et al. (1988) GMDH can produce exceedingly complex models. As an example, if the model has *r* layers based upon *m* regressors, the final polynomial representing the model would have degree $2^{r}$ with the number of terms being

$\left( \frac{2^{r}+m}{m} \right)$ (A14)

So, with four layers and six regressors within the input vector, the GMDH polynomial presents a degree of 16 with 74,613 terms. The partial description coefficients are determined by the regression method of least squares (Anastasakis & Mort, 2001). The data selected that forms the training set is used for the determination of the coefficients (Duffy & Franklin, 1975). When the regressors within the input vector are well defined, the coefficient estimates will be accurate, but if the regressors are ill-defined, then the coefficients will be subject to bias (Anastasakis & Mort, 2001). Real world data though is normally ill-defined and thus the coefficient estimates for the partial descriptions will be subject to bias. Ivakhnenko and Zholnarskij (1992) identify the method of instrumental variables as a potential replacement for least squares delivering estimates with less bias.

The average error in estimating the PD coefficients is illustrated mathematically (Madala & Ivakhnenko, 1994).

Let *b* denote the desired value and *y* the estimated output value for the PD under consideration. $N_{A}$ is the set of training data. The output errors are given by

$e_{q}= y_{q}- b_{q};q \in N_{A}$ (A15)

For the input vector, the total squared error is

$E= \sum_{q\in N_{A}} e_{q}^{2}$ (A16)

This is the average error minimised in coefficient estimation.

Each layer that forms the neural network details Madala and Ivakhnenko (1994) contains groups of neurons that link to neurons within the following layer. The PD coefficients at each neuron being estimated through minimising error *E*. The selection criterion is used to assess whether each neuron in each layer will be accepted or rejected. If accepted, the neuron is switched on allowing its output to form an input in the following layer. The process continues throughout the network until the most suitable output representative of the model is found by the selection criterion. The PD most frequently used is the second order polynomial, but there are alternatives (Anastasakis & Mort, 2001). Additional mathematical expressions that can be used within Parametric GMDH include linear and cubic equations (Farzi, 2008). MIA can also utilise transfer functions details (Kondo & Ueno, 2012) within each neuron simultaneously with the function that delivers the smallest mean square error being selected for that given neuron. Transfer functions include –

Sigmoid Function: $z= \frac{1}{(1+ e^{-y})}$ (A17)

Radial Basis Function: $z= e^{-y^{2}}$ (A18)

Tangent Function $z=tan(y)$ (A19)

## Appendix B

Take a given training set composed of $n$ data points {${x_{i}, y_{i}\}}_{i=1}^{n}$ , input data $x_{i}\in\mathbb{R}^{n}, p$ being the total number of data patterns. Output $y_{i}\mathbb{\in R}$.

The SVM approximation of the function is presented in the form

$y\left( x \right)= \boldsymbol{w}^{T}\emptyset\left( x \right)+b$ (B1)

with $\emptyset(x)$ the high dimensional feature spaces being nonlinearly mapped from the input space $\boldsymbol{x}$. In LSSVM for estimating the function, the formulation of an optimisation problem is presented (Suykens et al., 2002)

min $J\left( w, e \right)= \frac{1}{2}\boldsymbol{w}^{T}\boldsymbol{w}+ \frac{\gamma}{2}\sum_{i=1}^{n} e_{i}^{2}$ (B2)

The following constraints apply

$y\left( x \right)= \boldsymbol{w}^{T}\emptyset\left( x_{i} \right)+b+ e_{i}, i=1, 2, \ldots, n$ (B3)

To obtain the solution, the Lagrange is constructed

$L\left( w, b, e, \alpha\right)=J\left( w, e \right)- \sum_{i=1}^{N} \alpha_{i}\{\boldsymbol{w}^{T}\emptyset\left( x_{i} \right)+b+ e_{i}- y_{i}\}$ (B4)

With Lagrange multipliers $\alpha_{i}$. The conditions that apply for optimality are equations 38 - 41

$\frac{\partial L}{\partial w}=0 \to w= \sum_{i=1}^{N} \alpha_{i}\emptyset(x_{i})$ (B5)

$\frac{\partial L}{\partial b}=0 \to\sum_{i=1}^{N} \alpha_{i}=0$ (B6)

$\frac{\partial L}{\partial e_{i}}=0 \to\alpha_{i}= \gamma e_{i}$ (B7)

$\frac{\partial L}{\partial\alpha_{i}}=0 \to\boldsymbol{w}^{T}\emptyset\left( x_{i} \right)+b+ e_{i}- y_{i}=0$ (B8)

for $i=1, 2, \ldots, n.$

Following the elimination of $e_{i}$ and $w$ the solution is presented by the linear equations

$\left( \begin{matrix} 0 & \boldsymbol{1}^{T} \\ \boldsymbol{1} & \emptyset{(x_{i})}^{T}\emptyset\left( x_{l} \right)+ \gamma^{-1}\boldsymbol{I} \end{matrix} \right)\left( \begin{matrix} b \\ a \end{matrix} \right)= \left( \begin{matrix} 0 \\ y \end{matrix} \right)$ (B9)

with $y=\left[ y_{1};\ldots;y_{n} \right], 1=\left[ 1;\ldots;1 \right], \alpha=\left[ \alpha_{1};\ldots;\alpha_{n} \right]$

Utilising Mercer’s theorem which sets the conditions for which a function can be considered a Kernel (Bhattacharyya, 2018), the Kernel function is defined as being

$K\left( x_{i}, x_{j} \right)= \emptyset{(x_{i})}^{T}\emptyset\left( x_{j} \right), i, j=1,2,\ldots, n$ (B10)

The LSSVM model for estimating functions is now illustrated as

$y\left( x \right)= \sum_{i=1}^{n} \alpha_{i}K\left( x_{i},x_{j} \right)+b$ (B11)

noting that $\alpha_{i}, b$ are the solutions of the linear system

Samsudin et al. (2011) notes that selecting the Kernel function $K(.,..)$ has several options. $K\left( x_{i}, x_{j} \right)$ is the Kernel function, with its value equal to the inner product of two vectors $\boldsymbol{x}_{i}$ and $\boldsymbol{x}_{j}$ within the feature space $\emptyset(x_{i})$ and $\emptyset(x_{j})$, such that $K\left( x_{i},x_{j} \right)= \emptyset\left( x_{i} \right)* \emptyset(x_{j})$.

Examples of Kernel functions include

Linear: $K\left( x_{i},x_{j} \right)= \boldsymbol{x}_{i}^{T}\boldsymbol{x}_{j}$

Polynomial: $K\left( x_{i},x_{j} \right)=({\gamma\boldsymbol{x}_{i}^{T}\boldsymbol{x}_{j}+r)}^{d}, \gamma>0$

Radial Basis Function: $K\left( x_{i},x_{j} \right)=\exp\left( -\gamma{ǁ\boldsymbol{x}_{i}-\boldsymbol{x}_{j}ǁ}^{2} \right), \gamma>0$

Sigmoid: $K\left( x_{i},x_{j} \right)=tanh(\gamma\boldsymbol{x}_{i}^{T}\boldsymbol{x}_{j}+r)$ (B12)

Kernel parameters $\gamma, r,$and $d$ require careful selection given they implicitly define the spatial framework of the high dimensional feature space $\emptyset\left( x \right),$ thereby controlling final solution complexity. Construction of the hybrid LSSVM with GMDH follows the procedure of Samsudin et al. (2011). The complete dataset is to be normalised prior to being separated into two disjoint sets covering training and testing. Sotelo (2017) explains that optimisation occurs through minimisation of the decision vector $\boldsymbol{w}$, with the scale of the input data influencing the optimal hyperplane. The recommendation is made that the data be standardised with a mean of zero and a variance of one.

## Appendix C

Using rainfall data $X(t)$ as an example and the approach of Wu and Huang (2009), decomposition of the data in terms of IMFs $g_{j}$

$x\left( t \right)= \sum_{j=1}^{m} g_{j}+r_{m}$ (C1)

with $r_{m}$ the reside of data $x(t)$, following extraction of $m$ IMFs. The IMFs are oscillatory functions, simple in structure with both amplitude and frequency that vary. IMFs have the following properties

1. Across the full length of each IMF when comparing the number of extrema and the number of zero-crossings, their difference must be either zero, or at most one. This is not to be confused with the numbers from $X(t)$ which could be significantly different.
2. At any random location selected within the data, the envelope defining the local maxima and the envelope defining the local minima, their mean is zero.

Local extrema are only used by the EMD through a sifting process, so for any data,

1. All local extrema, both maxima, and minima, are to be identified, connecting all extrema with a cubic spline thus forming two envelopes.
2. The first component $A$ is obtained as the difference between the data and local mean of the envelope dyad.
3. Let $A$ now be treated as the data repeating steps a and b as often as necessary to deliver symmetric envelopes with respect to zero mean, with the final $A$ redefined as $g_{j}$.

The sifting is complete when the residue $r_{m}$ depicts a monotonic function preventing extraction of any further IMFs (Wu & Huang, 2009). The gradient of the residue illustrates the trend within the data which is very useful when viewing historic rainfall data. Before incorporating the additional details of the EEMD, it is useful to review a few important EMD properties (Wu & Huang, 2009):

1. Being based upon local data characteristics, the adaptive data analysis method effectively captures nonlinear, non-stationary oscillations with greater effect.
2. Any white noise only series or fractional Gaussian, EMD is equivalent to a dyadic filter bank.
3. Intermittent data can compromise the dyadic property.
4. A reference scale that is uniformly distributed could be obtained through the addition of noise enabling the compromised dyadic property to be repaired by EMD.
5. There is no correlation between corresponding IMFs depicting differing noise series. As such, cancellation of the means of corresponding IMFs presenting different white noise series is likely.

With this information clear and present, the EEMD features

1. The targeted data is augmented with a white noise series.
2. The data featuring the additional white noise is decomposed into IMFs.
3. Steps 1 and 2 are continually repeated with the addition of a different series of white noise on each occasion.
4. The ensemble means pertaining to the corresponding IMFs based upon the decompositions are obtained as the final result.

## Appendix D

(Refer as an example to *A wavelet tour of signal processing* by Stéphane Mallet, 1999 for an in-depth mathematical explanation.)

A function $\psi(x)$ in satisfying these conditions

1. $E=\int_{-\infty}^{\infty} |\psi\left( t \right)|^{2}dt<\infty$
2. $C_{\psi}=\int_{-\infty}^{\infty} \frac{|\hat{\psi}\left( f \right)|^{2}}{f}$ d$f \equiv C_{\psi}<\infty$

is a wavelet with condition 1. possessing finite energy. If $\hat{\psi}(f)$ is the Fourier transform of $\psi(t)$, then condition 2 is a necessary requirement. The implication being that $\hat{\psi}=0$. This is the admissibility condition, and $C_{\psi}$ the admissibility constant.

A wavelet family is a group of functions acquired by translating and dilating a wavelet graph. The mother wavelet $\psi(x)$ is composed of functions $\psi_{a,\tau}(x)$ such that

$\psi_{a,\tau}\left( x \right)=\frac{1}{\sqrt{a}}\psi\left( \frac{x-\tau}{a} \right)$ (D1)

with $\tau$ is the translation or centre of $\psi_{a,\tau}$ and $a$ is the dilation parameter.

A function $f(x)$ with CWT, as introduced by Morlet in 1984 being defined as

$Wf\left( a,\tau\right)=\int_{-\infty}^{\infty} f(x)\psi_{a,\tau}(x) dx$ (D2)

with inverse transform

$f\left( x \right)=\frac{1}{C_{\psi}}\iint_{-\infty}^{\infty} \frac{1}{|a|^{\frac{3}{2}}}Wf\left( a,\tau\right)\psi_{a,\tau}\left( x \right) da d\tau$ (D3)

The wavelet transform of the function $f$ is the convolution with the conjugate wavelet (Mallat, 1999):

$Wf\left( \tau,a \right)=\frac{1}{\sqrt{a}}\int_{-\infty}^{\infty} f(t)\psi^{*}\left( \frac{t-\tau}{a} \right)dt$ (D4)

where the mother wavelet $\psi\left( t \right)=\pi^{-\frac{1}{4}}e^{-\frac{t^{2}}{2}}(e^{-i\omega_{0}t}-e^{-\frac{\omega_{0}^{2}}{2}})$ (D5)

with $\omega_{0}=\sqrt{\frac{2}{ln2}}$ and $i=\sqrt{-1}$ (Russell and Han, 2016).

It is important to note that if using the CWT for pre-processing the graph of the monthly rainfall data before supplying this information to GMDH, the CWT stores changes in $f(x)$ allowing both compression and removal of noise.

In the case of the DWT the values of $a$ and $\tau$ are limited. As the signal is represented discretely, numerical computations are required for the integrals that define the coefficients. In taking a mother wavelet, to obtain an orthogonal family of wavelets select $a= a_{0}^{m}$ and $\tau=n\tau_{0}$, with $m$ and $n$ integers, $a_{0}$ is a dilation parameter $>1$, and $\tau_{0}$ a translation parameter $>0$. The DWT is now presented as

$Wf\left( m,n \right)=\left\langle\psi_{m,n},f \right\rangle=\int_{-\infty}^{\infty} \psi_{m,n}\left( x \right)f\left( x \right)dx$ (D6)

with

$\psi_{m.n}\left( x \right)=2^{-m/2}\psi\left( \frac{x-n2^{m}}{2^{m}} \right)$ (D7)

The inverse transform being given by

$f\left( x \right)=\sum_{m,n} \psi_{m,n}\left( x \right)Wf(m,n)$ (D8)

## Appendix E

In providing a mathematical explanation of the integration of the LM algorithm with GMDH (and SVD for the initial guess), some background terminology is first introduced. With a given data set $d(t_{i},y_{i})$ and a model function $\emptyset(\boldsymbol{x};t_{i})$, the difference of the functions is obtained with $r_{i}\left( x \right)=\emptyset\left( \boldsymbol{x};t_{i} \right)-y_{i}$ noting that $y_{i}$ is the $y$ component of data point $t_{i}$ (Croeze et al., 2012). Thus, defining the objective function (cost function) as it applies in problems of least squares:

$f\left( x \right)=\frac{1}{2}\sum_{i=1}^{m} r_{i}^{2}(x)$ (E1)

Minimising $f(x)$ allows the parameters to be found that match most accurately the model to the observed data. Each residual $r_{i}$is a smooth function from $\mathbb{R}^{n}$ to $\mathbb{R}$. The residual vector of $m$ components is given by:

$\boldsymbol{r}\left( x \right)=({r_{1}\left( x \right),r_{2}\left( x \right),\ldots,r_{m}\left( x \right))}^{T}$ (E2)

This allows eq. (E1) to be rewritten using the residual vector:

$f\left( x \right)= \frac{1}{2}ǁ\boldsymbol{r}(x)ǁ^{2}$ (E3)

The gradient of the residual vector $\boldsymbol{r}(x)$ is required when calculating the gradient of the objective function $f(x)$. The Jacobian $\boldsymbol{J}(x)$ is a matrix with elements all $\nabla r_{i}(x)$. Both the gradient and the Hessian of the objective can be expressed explicitly in terms of the Jacobian:

$\nabla f\left( x \right)=\sum_{i=1}^{m} \boldsymbol{r}_{i}\left( x \right)\nabla\boldsymbol{r}_{i}\left( x \right)=\boldsymbol{J}\left( x \right)^{T}\boldsymbol{r}(x)$ (E4)

$\nabla^{2}f\left( x \right)=\sum_{i=1}^{m} \nabla\boldsymbol{r}_{i}(x)\nabla\boldsymbol{r}_{i}(x)^{T}+\sum_{i=1}^{m} \boldsymbol{r}_{i}\left( x \right)\nabla^{2}\boldsymbol{r}_{i}\left( x \right)=\boldsymbol{J}(x)^{T}\boldsymbol{J}\left( x \right)+\sum_{i=1}^{m} \boldsymbol{r}_{i}(x)\nabla^{2}\boldsymbol{r}_{i}(x)$ (E5)

It is noted that it is a requirement of the Hessian Matrix to be positive definite for least squares problems. In the instance when both the residual and the solution are extremely close, $\nabla^{2}f(x)$ approximation can be made using only the first term. This approximation is utilised in both the GN and LM methods (Croeze et al., 2012; Lourakis, 2005). Developed by Levenberg (1944) and Marquardt (1963) as a method for solving nonlinear least squares problems that brings together the methods of GD and GN (Lourakis, 2005). Given that both methods complement each other in their advantages, Levenberg proposed the algorithm (Ranganathan, 2004)

$\boldsymbol{x}_{i+1}=\boldsymbol{x}_{i}-(\boldsymbol{H}+{\lambda\boldsymbol{I})}^{-1}\nabla f(\boldsymbol{x}_{i})$ (E6)

noting that $\boldsymbol{H}$ is the Hessian matrix being evaluated at $\boldsymbol{x}_{i}$ and $\lambda$ is the damping parameter.

The disadvantage of this algorithm being, should $\lambda$ be large, then $\boldsymbol{H}$ is not used. This problem was solved by Marquardt when the identity matrix in eq. (E6) was replaced by the diagonal of $\boldsymbol{H}$ which delivered the LM algorithm (Ranganathan, 2004).

$\boldsymbol{x}_{i+1}=\boldsymbol{x}_{i}-(\boldsymbol{H}+\lambda diag{\left[ \boldsymbol{H} \right])}^{-1}\nabla f(\boldsymbol{x}_{i})$ (E7)

It is noted that the inverse is often achieved by iplementing SVD (Ranganathan, 2004).

An equal but alternative representation is presented utilising the Jacobian, as detailed by Manfre (2021):

$\left( \boldsymbol{J}^{T}\boldsymbol{J}+\lambda\boldsymbol{I} \right)\alpha_{i}=\boldsymbol{J}^{T}[{\hat{\boldsymbol{y}}}_{i}-f\left( \boldsymbol{a}_{n} \right)]$ (E8)

with ${\hat{\boldsymbol{y}}}_{i}$ the known output, and the Jacobian Matrix (Transtrum et al., 2011) is the matrix of the residuals with respect to the parameters (p. 1).

Step size can be calculated by rearranging eq. (E8) to deliver

$\alpha_{i}=(\boldsymbol{J}^{T}\boldsymbol{J}+{\lambda\boldsymbol{I})}^{-1}\boldsymbol{J}^{T}[f\left( \boldsymbol{x},\boldsymbol{a} \right)-{\hat{\boldsymbol{y}}}_{i}]$ (E9)

References

Bhattacharyya S (2018) Support vector machine: Kernel trick; Mercer’s theorem. Towards Data Science

<https://towardsdatascience.com/understanding-support-vector-machine-part-2-kernel-trick-mercers-theorem-e1e6848c6c4d>

Croeze A, Pittman L, Reynolds W (2012) Solving nonlinear least-squares problems with the Gauss-Newton and Levenberg-Marquardt methods. <https://www.math.lsu.edu/system/files/MunozGroup1%20-%20Presentation.pdf>

Duffy JJ, Franklin MA (1975) A learning identification algorithm and its application to an environmental system. IEEE Trans Syst Man Cybern smc-5(2):226–240

Farzi S (2008) A new approach to polynomial networks based on genetic algorithm. World Acad Sci Eng Technol 2(8):2700–2707

GMDH (2022) Group method of data handling. Retrieved from <http://www.gmdh.net/>

Ivakhnenko AG, Zholnarskij AA (1992) Estimating the coefficients of polynomials in parametric GMDH algorithms by the improved instrumental variables method. J Autom Inf Sci 25(3):25–32

Jiřina M, Jiřina M Jr (2013) GMDH method with genetic selection algorithm and cloning. Neural Netw World 1(2013):451–464

Kondo T, Ueno J (2012) Feedback GMDH-type neural network and its application to medical image analysis of liver cancer. 42nd ISCIE international symposium on stochastic systems theory and its applications, 81–82. <https://www.researchgate.net/publication/289143779_Feedback_GMDHtype_Neural_Network_and_Its_Application_to_Medical_Image_Analysis_of_Liver_Cancer>

Levenberg K (1944) A method for the solution of certain non-linear problems in least squares. Q Appl Math 2(2):164–168

Lourakis MIA (2005) A brief description of the Levenberg-Marquardt algorithm implemented by levMar.

<https://www.semanticscholar.org/paper/A-Brief-Description-of-the-Levenberg-Marquardt-by-Lourakis/a92844ea0726e0ebb3fdf99b070d1e8e289eb97f>

Mallat S (1999) A wavelet tour of signal processing. Academic Press, London

Manfre D (2021) Towards data science: the interesting world of nonlinear regressions. Retrieved from https://towardsdatascience.com/the-interesting-world-of-nonlinear-

regressions-eb0c405fdc97

Marquardt D (1963) An algorithm for the least-squares estimation of nonlinear parameters. SIAM J Appl Math 11(2):431–441

Nikolaev NY, Iba H (2003) Polynomial harmonic GMDH learning networks for time series modelling. Neural Netw 16(2003):1527–1540. https://doi.org/10.1016/S0893-6080(03)00188-6.(psu.edu)

Pham DT, Liu X (1994) Modelling and prediction using GMDH networks of Adalines with nonlinear pre-processors. Int J Syst Sci 25(11):1743–1759. https://doi.org/10.1080/00207729408949310

Ranganathan A (2004) The Levenberg-Marquardt algorithm. https://www.researchgate.net/publication/2877846_The_Levenberg-Marquardt_Algorithm

Russell B, Han J (2016) Jean Morlet and the continuous wavelet transform. CREWES Res Rep 28(2016):115

Singer Y (2016) Lecture 9, AM221 Advanced optimisation. Harvard University, Cambridge

Sotelo D (2017) Effect of feature standardisation on linear support vector machines. Towards data science. https://towardsdatascience.com/effect-of-feature-standardization-on-linear-support-vector-machines-13213765b812

Suykens JAK, Van Gestel T, De Brabanter J, De Moor B, Vandewalle J (2002) Least squares support vector machines. World Scientific Publishing, Singapore. https://sciarium.com/file/158618/

Zinn-Bjorkman L, Harp DR, Vesselinov V (2011) Numerical optimisation using the Levenberg-Marquardt algorithm. https://doi.org/10.13140/RG.2.2.11253.01760
